# *FAST-1* antisense RNA epigenetically alters *FXN* expression

**DOI:** 10.1038/s41598-018-35639-2

**Published:** 2018-11-21

**Authors:** Hajar Mikaeili, Madhavi Sandi, Aurélien Bayot, Sahar Al-Mahdawi, Mark A. Pook

**Affiliations:** 1 Division of Biosciences, Department of Life Sciences, College of Health & Life Sciences, and Synthetic Biology Theme, Institute of Environment, Health & Societies, Brunel University London, Uxbridge, United Kingdom; 2 Present Address: Mitochondrial Biology Group, CNRS UMR 3691, Departement of Cell Biology and Infection, Institut Pasteur, Paris, France

## Abstract

Friedreich ataxia (FRDA) is a multisystem genetic disorder caused by GAA repeat expansion mutations within the *FXN* gene, resulting in heterochromatin formation and deficiency of frataxin protein. Elevated levels of the *FXN* antisense transcript (*FAST-1*) have previously been detected in FRDA. To investigate the effects of *FAST-1* on the *FXN* gene expression, we first stably overexpressed *FAST-1* in non-FRDA cell lines and then we knocked down *FAST-1* in FRDA fibroblast cells. We observed decreased *FXN* expression in each *FAST-1* overexpressing cell type compared to control cells. We also found that *FAST-1* overexpression is associated with both CCCTC-Binding Factor (CTCF) depletion and heterochromatin formation at the 5′UTR of the *FXN* gene. We further showed that knocking down *FAST-1* in FRDA fibroblast cells significantly increased *FXN* expression. Our results indicate that *FAST-1* can act *in trans* in a similar manner to the *cis-*acting *FAST-1* overexpression that has previously been identified in FRDA fibroblasts. The effects of stably transfected *FAST-1* expression on CTCF occupancy and heterochromatin formation at the *FXN* locus suggest a direct role for *FAST-1* in the FRDA molecular disease mechanism. Our findings also support the hypothesis that inhibition of *FAST-1* may be a potential approach for FRDA therapy.

## Introduction

Friedreich ataxia (FRDA), the most prevalent inherited ataxia, is an autosomal recessive neurodegenerative disorder, primarily affecting the nervous system and the heart. This progressive disease is characterized by limb and gait ataxia, dysarthria, hypertrophic cardiomyopathy and skeletal abnormalities^1^. Most patients are homozygous for expanded GAA triplet repeat within the first intron of the frataxin (*FXN*) gene^2^. Unaffected individuals have up to ~40 triplets, whereas in FRDA patients the number of GAA repeats can be from 70 to 1700^3,4^. The expanded repeats cause a severe deficiency of transcriptional initiation of the *FXN* gene that ultimately leads to reduction of the essential mitochondrial protein frataxin^5,6^. Frataxin (FXN) is a nuclear encoded, highly conserved protein which is involved in iron-sulfur cluster (ISC) biosynthesis and regulating mitochondrial iron transport and respiration^7,8^. Although the exact molecular mechanism of *FXN* gene silencing is still unknown, accumulating evidence indicates that epigenetic changes play a crucial role in inhibition of *FXN* transcription. Work with transgenic mice showed that it is the intrinsic property of the expanded GAA repeat that causes heterochromatin formation to exert its epigenetic gene silencing effect^9^. FRDA alleles have been shown to be enriched for molecular signatures of heterochromatin including histone H3 and H4 deacetylation, histone trimethylation (H3K9me3 and H3K27me3), CpG methylation and non-coding RNA transcription^10–14^. Investigating DNA methylation profiles of the *FXN* gene in FRDA cell models, human and transgenic mouse tissues demonstrated elevated CpG methylation levels upstream of the expanded repeats. The amount of DNA methylation correlates with the extent of GAA expansion, phenotype severity and age of disease onset^12,15,16^. Interestingly, no changes in DNA methylation have been detected in the 5′ untranslated region (UTR) of the *FXN* gene. Enrichment of repressive chromatin marks at the *FXN* promoter, upstream and downstream GAA regions have been reported in lymphoblastoid and fibroblast cells^14,17^ and in FRDA human and transgenic mouse brain and heart tissues^12^. A number of studies have demonstrated that reversing epigenetic changes via administration of histone deacetylase inhibitors (HDACi) can restore *FXN* transcription in FRDA^10,18^. These results further support the hypothesis that transcriptional silencing is due to epigenetic aberrations. In FRDA, heterochromatin encompasses the *FXN* transcription start site (*FXN-TSS*) and silences the promotor activity^6^. In addition, severe depletion of the chromatin insulator protein CTCF has been identified at the 5′ untranslated region (UTR) of the *FXN* gene in FRDA patients. An antisense transcript named *FAST-1* (*FXN* Antisense Transcript – 1), whose sequence overlaps with the CTCF binding site, has also been discovered. *FAST-1* expression is significantly increased in FRDA and is associated with the severe CTCF depletion and heterochromatin formation in the 5′UTR of the *FXN* gene^14,19^. Natural antisense transcripts (NATs) have long been described as ‘junk DNA’ or transcriptional noise due to their low expression and unknown function. However, in recent years, antisense transcripts have emerged as key regulators of gene expression in an epigenetic manner^20–23^.

Literature supporting the notion that antisense transcripts are involved in heterochromatin formation and the regulation of their partner mRNA expression inspired us to further investigate the characteristics of *FAST-1*. We first identified a full-length *FAST-1* transcript with a total length of 523 bp in size containing a poly (A) tail. Mapping the 3′ and 5′ ends of the *FAST-1* transcript onto the genome showed that *FAST-1* transcription overlaps with the *FXN*-TSS and CTCF binding site in the 5′UTR of the *FXN* gene. Therefore, we decided to investigate potential effects of altered *FAST-1* expression on *FXN* expression in three different types of cell lines. We report that *FAST-1* overexpression is consistently associated with reduced CTCF occupancy, heterochromatin formation and decreased *FXN* expression. We also show that knocking down *FAST-1* expression results in increased *FXN* expression in FRDA fibroblast cells, thereby revealing *FAST-1* to be a potential FRDA therapeutic target.

## Results

### Identification of *FAST-1* by rapid amplification of cDNA ends

**Figure 1:**
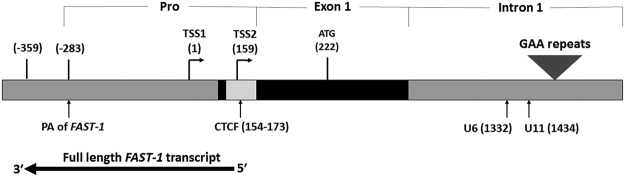
The 5′ end of *FXN* gene showing the region corresponding to the full length *FAST-1* transcript. It also contains a polyadenylation signal (PA) located between −283 to −288. The 5′-end of *FAST-1* coincides with the CTCF binding site in the *FXN* 5′UTR. Pro = *FXN* promoter, TSS = transcription start site, U6 and U11 = CpG sites.

To determine the exact size and location of *FAST-1*, 5′ and 3′ rapid amplification of cDNA ends (RACE) experiments were performed with total RNA and poly(A) + RNA. Mapping the 5′ and 3′ ends of *FAST-1* transcript onto the genome precisely localised them to nucleotides + 164 and −359 of the *FXN* gene, respectively, and the total length of *FAST-1* was found to be 523 bp in size. A poly (A) signal was also identified in the *FAST-1* sequence at *FXN* nucleotide positions −283 to −288 (Fig. 1).

### Stable overexpression of *FAST-1* in non-FRDA cells

**Figure 2:**
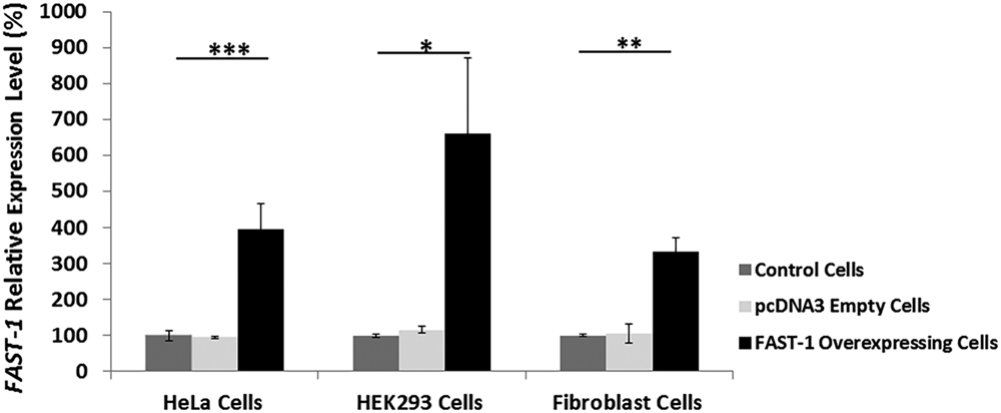
Relative *FAST-1* expression in *FAST-1* overexpressing cells. *FAST-1* overexpression was confirmed in *FAST-1* transfected cells compared to non-transfected and empty-vector-transfected control cells. Data were normalized to the mean *FAST-1* level of non-transfected control cells taken as 100%. (n = 12, *FAST-1* overexpressing HeLa and HEK293 clones), (n = 3, *FAST-1* overexpressing fibroblast clones), *p < 0.05, **p < 0.01, ***p < 0.001, Bars represent SEMs.

To assess the effect of *FAST-1* on *FXN* expression, we generated three non-FRDA cell lines - HeLa, HEK293 and fibroblast - that stably overexpress *FAST-1* cDNA under the control of a CMV promoter. Twelve stable *FAST-1* overexpressing HeLa cell lines, twelve stable *FAST-1* overexpressing HEK293 cell lines and three stable *FAST-1* overexpressing fibroblast cell lines were developed. To confirm *FAST-1* overexpression in *FAST-1* transfected cells, qRT-PCR measurements were performed, using primers designed to detect human-specific frataxin antisense transcript, *FAST-1*. Analysis of qRT-PCR measurement confirmed a very significant increase of *FAST-1* expression in transfected HeLa cells (396%, P < 0.001), transfected HEK293 cells (660%, P < 0.05) and transfected fibroblast cells (332%, P < 0.01) compared with non-transfected control cells (Fig. 2). No such differences were detected with the empty vector-transfected cells.

### Overexpression of *FAST-1* reduces the *FXN* mRNA and protein expression

**Figure 3:**
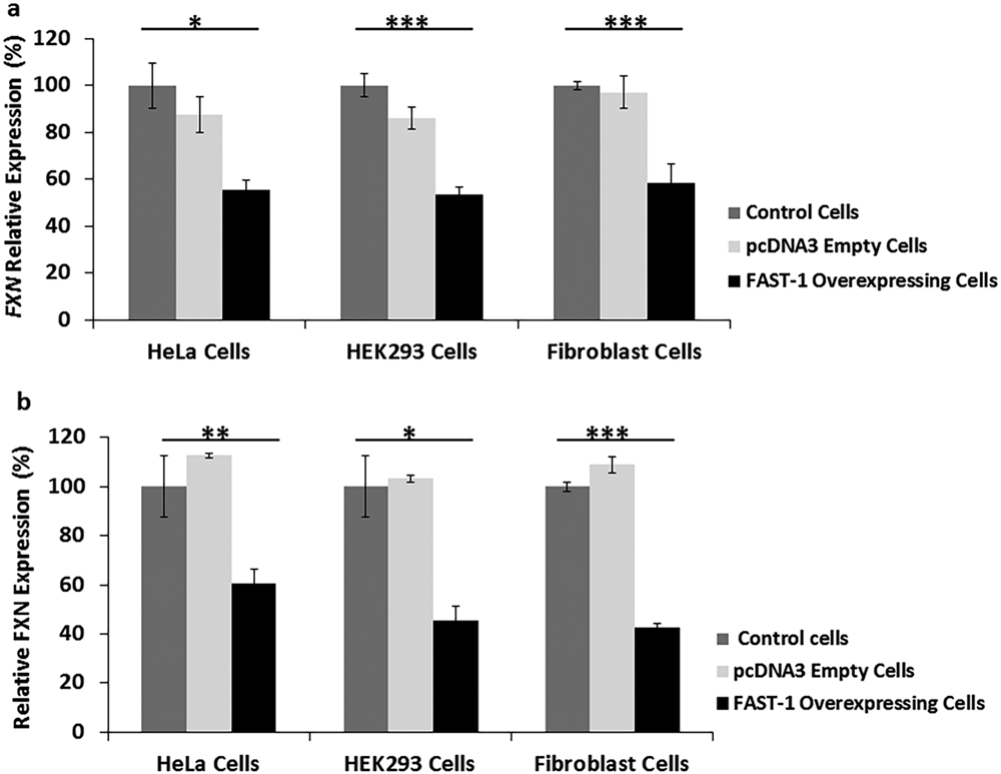
Frataxin expression levels in *FAST-1* overexpressing cells. (**a**) qRT–PCR analysis of *FXN* mRNA levels in *FAST-1* overexpressing cells. Data were normalized to the mean *FXN* level of non-transfected control cells taken as 100%. (**b**) Dipstick immunoassay of human frataxin protein. Data were normalized to the mean *FXN* level of non-transfected control cells taken as 100%. (n = 12, *FAST-1* overexpressing HeLa and HEK293 clones), (n = 3, *FAST-1* overexpressing fibroblast clones), *p < 0.05, **p < 0.01, ***p < 0.001, Bars represent SEMs.

Analysis of *FAST-1* overexpressing cells revealed a very significant reduction of *FXN* mRNA in the *FAST-1* overexpressing HeLa cells (45%, P < 0.05), HEK293 cells (47%, P < 0.001) and fibroblast cells (42%, P < 0.001) compared with non-transfected control cells. There was no significant difference detected between the empty vector-transfected and non-transfected control cells (Fig. 3a).

To determine the levels of human frataxin expression in the *FAST-1* overexpressing cells, frataxin protein expression levels were measured by lateral flow immunoassay with the Frataxin Protein Quantity Dipstick assay kit. Analysis of *FAST-1* overexpressing cells revealed that frataxin expression levels were significantly decreased to (40%, P < 0.01) in the *FAST-1* overexpressing HeLa cells, HEK293 cells (55%, P < 0.05) and fibroblast cells (58%, P < 0.001) compared with non-transfected control cells (Fig. 3b). No such differences were detected with the empty vector-transfected cells.

### *FAST-1* copy number associated gene expression changes

**Figure 4:**
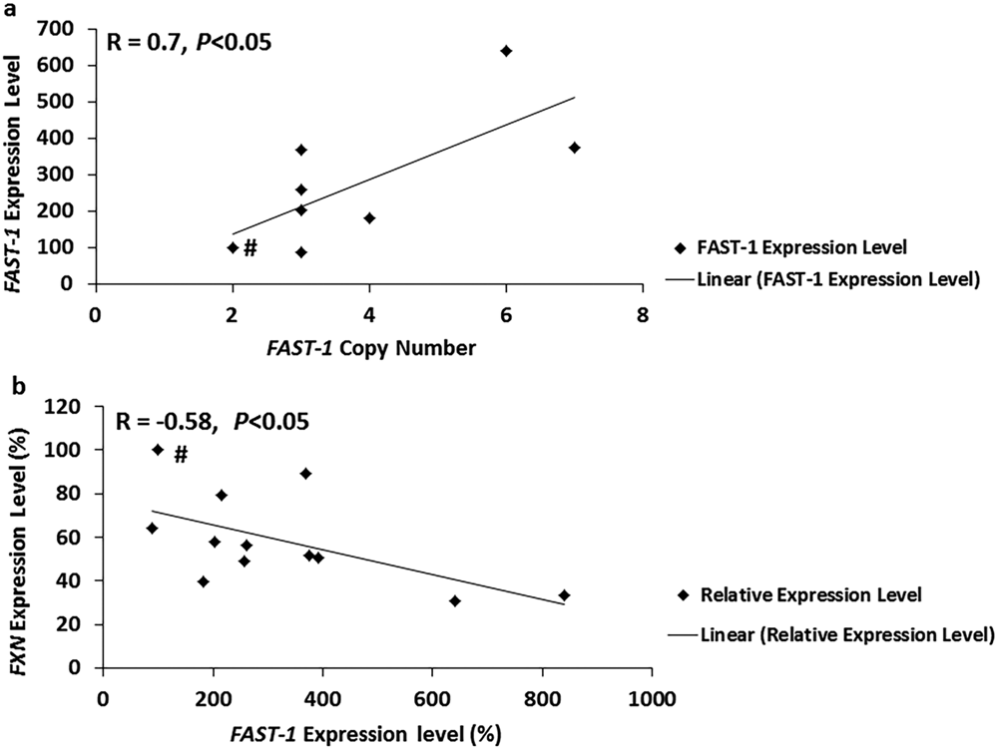
(**a**) F*AST-1* copy number in *FAST-1* overexpressing clones. TaqMan copy number assays were applied in duplicates. Non-transfected HeLa cells known to have two copies of *FAST-1* were used as the calibrator samples. (n = 7, *FAST-1* overexpressing HeLa clones). (**b**) Scatter plot of *FXN* mRNA expression level versus *FAST-1* expression in *FAST-1* overexpressing HeLa cells. There was a significant negative correlation between *FAST-1* expression and *FXN* expression in *FAST-1* overexpressing HeLa cells. (n = 11, *FAST-1* overexpressing HeLa clones).

The *FAST-1* copy number was investigated in *FAST-1* overexpressing HeLa cell genomic DNA samples using TaqMan real-time PCR, compared to non-transfected HeLa cells that contain two copies of the *FXN* locus. To assess the reliability of each copy number call, the results were analysed by Applied Biosystems CopyCaller™ Software v.2.0. Seven out of twelve *FAST-1* overexpressing HeLa cell lines, together with the endogenous non-transfected HeLa cell control, had acceptable confidence values and absolute zero score to pass the test. The results indicated that *FAST-1* copy number is positively correlated with increased *FAST-1* expression (R = 0.7, P < 0.05) (Fig. 4a), which in turn is negatively correlated with *FXN* expression by the analysis of eleven transfected HeLa cell lines and the endogenous non-transfected HeLa cell control (R = −0.58, *P* < 0.05) (Fig. 4b). This suggests a concentration-dependent molecular mechanism of action for *FAST-1*, rather than a potential threshold effect.

### *FAST-1* overexpressing HeLa cells show reduced occupancy of CTCF and marks of heterochromatin formation at the FXN 5′UTR locus

**Figure 5:**
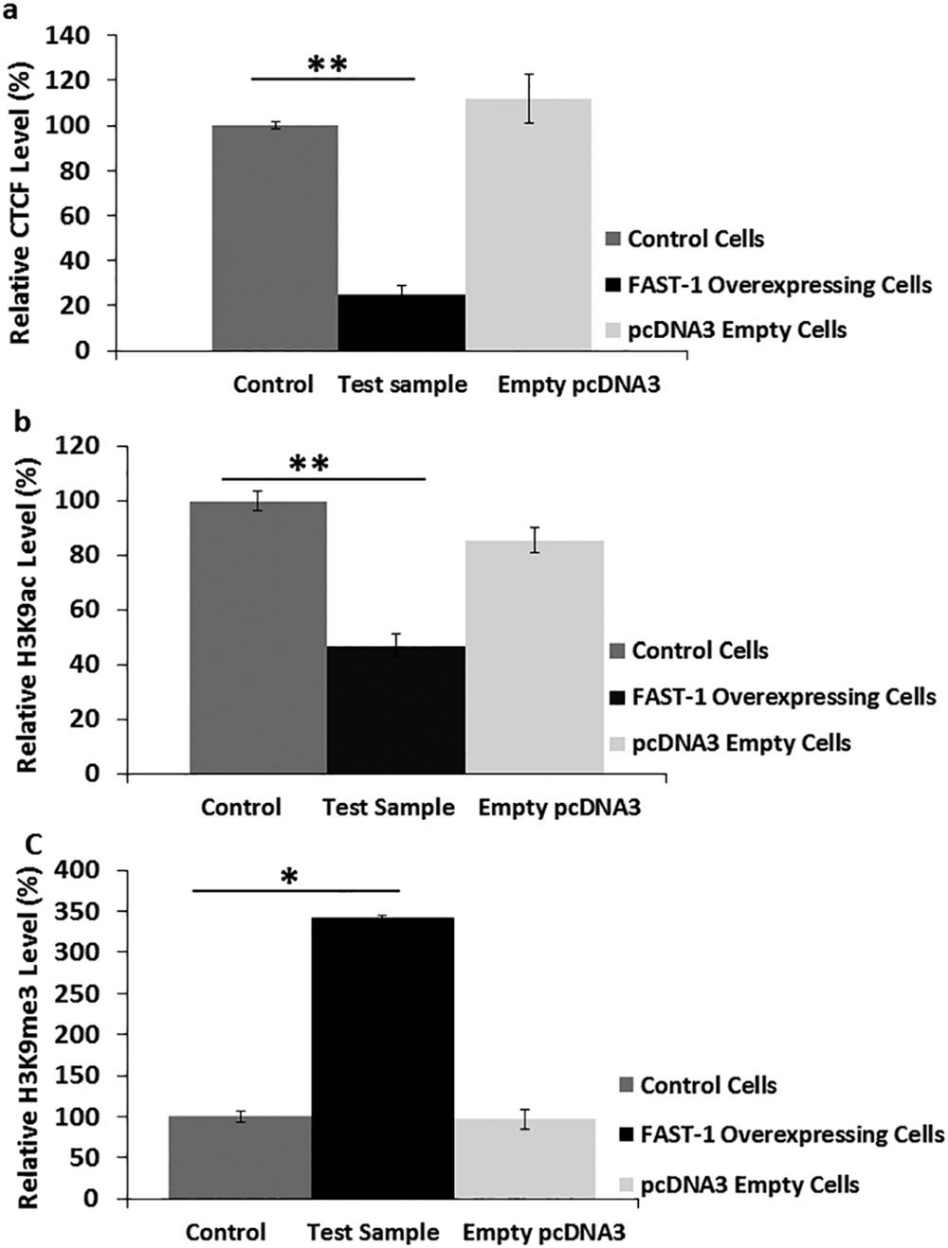
(**a**) CTCF analysis. ChIP analysis showing the relative CTCF occupancy in the *FXN* 5′UTR of *FAST-1* overexpressing HeLa cells. (**b**,**c**) Analysis of histone modifications in *FAST-1* overexpressing HeLa cells. ChIP quantitative PCR results for the *FXN* 5′UTR amplified regions are represented as the relative amount of immunoprecipitated DNA compared with ‘input’ DNA, having taken negligible −Ab control values into account. Mean values from the non-transfected Hela control cells are 100%. The data were from three independent chromatin preparations, with each experiment done in triplicate. (n = 3, *FAST-1* overexpressing HeLa clones). *p < 0.05, **p < 0.01, Bars represent SEMs.

It has previously been reported that CTCF depletion results in increased *FAST-1* transcription^14^. To elucidate the relationship between CTCF and *FAST-1*, CTCF occupancy was measured in *FAST-1* overexpressing HeLa cells. Three different *FAST-1* overexpressing HeLa clones, which showed the lowest level of *FXN* expression, empty vector-transfected cells and non-transfected control HeLa cells, were utilized in this experiment. CTCF ChIP analysis revealed that CTCF occupancy was decreased sharply at the 5′UTR of the *FXN* gene. In addition, comparison of CTCF occupancy in empty transfected-vector and non-transfected HeLa control cells did not show a similar reduction (Fig. 5a). We also investigated acetylated and trimethylated histone H3K9 modifications by ChIP analysis at the *FXN* 5′UTR region. The ChIP results were normalized to the ‘input’, and negative control antibody values were taken into account. ChIP assay results showed significantly decreased acetylation of H3K9 (Fig. 5b) and enrichment of H3K9me3 (Fig. 5c) at the *FXN* 5′UTR region in *FAST-1* overexpressing HeLa cells compared to non-transfected control cells. There were no statistically significant differences in H3K9 acetylation and trimethylation levels between the empty vector-transfected and non-transfected control cells.

### *FXN* gene DNA methylation levels are altered in *FAST-1* overexpressing fibroblast cells

**Figure 6:**
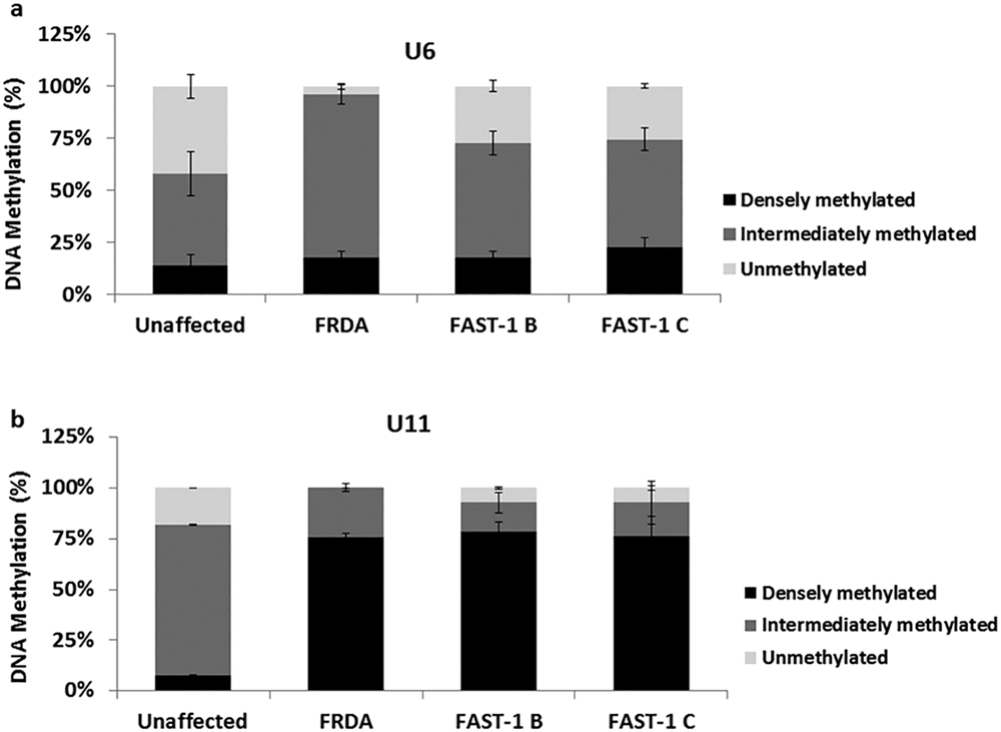
DNA methylation levels in *FAST-1* overexpressing fibroblast cells. MethylScreen analysis of CpG site (**a**) U6 and (**b**) U11 in the *FXN* upstream GAA repeat region of DNA from FRDA, unaffected control and *FAST-1* overexpressing fibroblast cells. (n = 2, *FAST-1* overexpressing fibroblasts).Bars represent SEMs.

It has been reported that CpG sites, designated U6 and U11, at the upstream GAA repeat region show elevated levels of DNA methylation in FRDA patients compared to controls^11,19^. Therefore, we chose to investigate the DNA methylation status at CpG U6 and U11 in two *FAST-1* overexpressing fibroblast cell lines (FAST-1B, FAST-1C), together with FRDA fibroblasts and unaffected control fibroblasts. Results from MethylScreen assays revealed a non-significant small increase in DNA methylation at CpG site U6, with DM values increasing from 14% in unaffected control fibroblasts to 18% and 23% in FAST-1 B and FAST-1 C, respectively (Fig. 6a). At CpG site U11, the proportion of densely methylated templates in *FAST-1* overexpressing fibroblasts significantly increased from 8% in unaffected controls to 79% (P < 0.01) and 76% (P < 0.001) in FAST-1 B and FAST-1 C, respectively, similar to the levels found in FRDA fibroblasts (Fig. 6b).

### Knocking down *FAST-1* in FRDA fibroblast cells increases FXN expression

**Figure 7:**
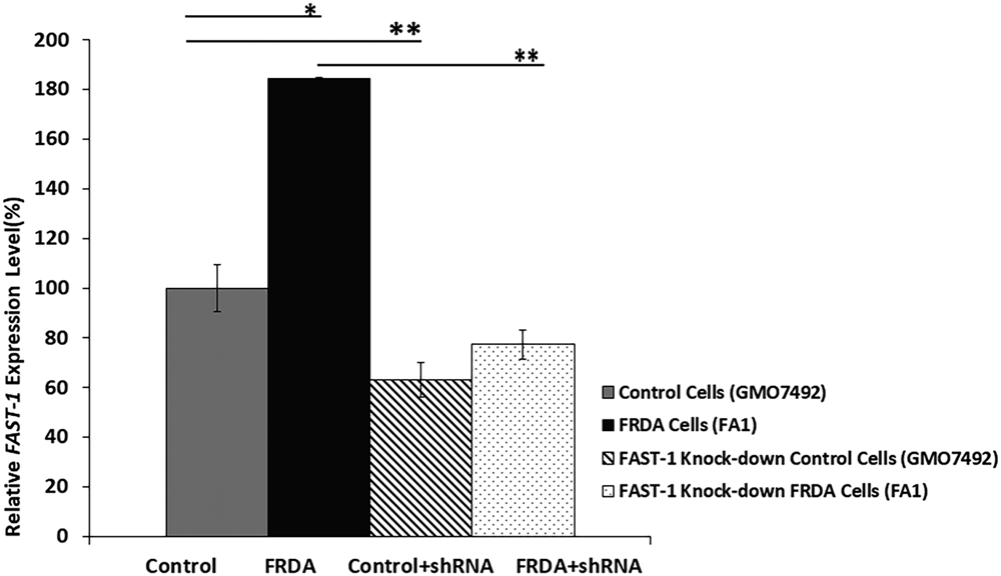
Quantitative RT–PCR analysis of *FAST-1* expression levels in FRDA fibroblasts and *FAST-1* knockdown fibroblast cells. qRT-PCR showed approximately twice as much *FAST-1* expression in FRDA cell lines versus non-FRDA controls. The efficacy of shRNA knockdown was assessed by qRT-PCR, demonstrating a significant reduction in the levels of *FAST-1* in both control fibroblast and *FAST-1* knockdown FRDA fibroblast cells. (n = 3). *p < 0.05, **p < 0.01, Bars represent SEMs.

**Figure 8:**
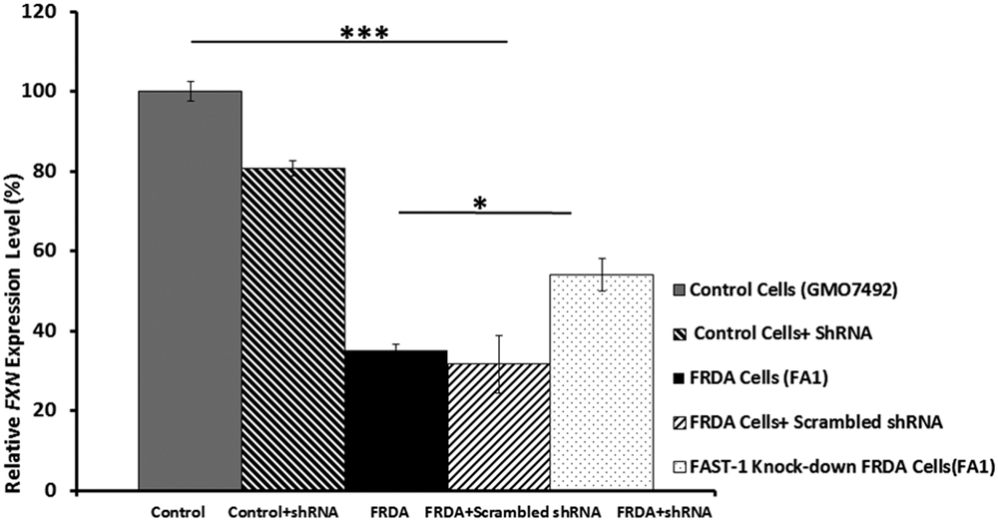
Quantitative RT–PCR analysis of *FXN* expression levels in control and *FAST-1* knockdown cell lines. Knocking down *FAST-1* in FRDA fibroblast cells increased *FXN* expression, but not to the level of control cells; however it did not significantly affect *FXN* expression in control cells. *FXN* expression in FRDA cells treated with scrambled shRNA remained unchanged. (n = 3). *p < 0.05, ***p < 0.001, Bars represent SEMs.

To investigate the effect of *FAST-1* silencing on the *FXN* expression in FRDA cells, FRDA and normal fibroblast cell lines were transduced with lentiviral (LV) vectors expressing shRNAs targeting *FAST-1* (pLKO.1-puro-CMV-tGFP). The efficacy of shRNA knockdown was assessed by qRT-PCR. Analysis of *FAST-1* expression levels in the *FAST-1* knockdown control and FRDA fibroblast cell lines revealed that *FAST-1* levels decreased to 63% (P < 0.01) and 43% (P < 0.01), respectively (Fig. 7). After confirming efficient *FAST-1* knockdown in FRDA fibroblast cells, *FXN* mRNA expression was measured in *FAST-1* knockdown cells. Our results demonstrated that the levels of *FXN* expression in *FAST-1* knockdown FRDA cells increased by 1.5-fold (*P* < 0.05) when compared to FRDA fibroblast cells. In contrast, *FXN* expression in normal fibroblast cells treated with LV *FAST-1* showed a non-statistically significant 0.8-fold decrease in the *FXN* expression. *FXN* expression remained unchanged in FRDA fibroblast cells treated with scrambled control shRNA (Fig. 8).

### Restoring aconitase activity following knockdown of *FAST-1*

**Figure 9:**
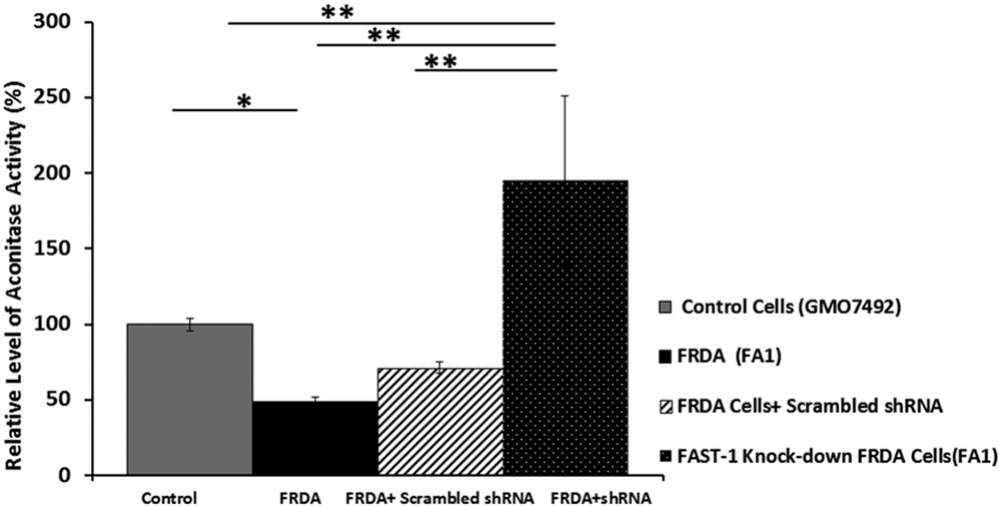
Aconitase activity levels in control and *FAST-1* knockdown cell lines. The experiment was performed in triplicate with values being calculated relative to citrate synthase activity. Aconitase activity in normal fibroblast cells was set at 100% all other samples were measured relative to these normal fibroblast cells. *p < 0.05, **p < 0.01, Bars represent SEMs.

As a biomarker of frataxin function within cells, aconitase activity was measured in *FAST-1* knockdown fibroblast cells 1 month after treatment with LV *FAST-1* using the Aconitase Assay Kit (Cayman). Normal FRDA fibroblast cells (GMO7492) and FRDA cells treated with scrambled shRNA were used as the controls. All data were calibrated to the mean of the aconitase activity in normal fibroblast cells, which was set to 100%. The aconitase activity in FRDA cells was determined to be 49% (*P* < 0.05) compared to normal control fibroblast cell levels. However, after knocking down *FAST-1* in FRDA fibroblast cells, aconitase activity significantly increased to a value of 195% (*P* < 0.01). No significant difference was detected in FRDA fibroblast cells treated with scrambled shRNA (Fig. 9).

## Discussion

Although it is more than 20 years since the identification of the genetic mutation underlying FRDA, the exact mechanisms by which GAA repeat expansions lead to *FXN* gene silencing are still not fully understood. However, several potential mechanisms have been proposed based on experimental evidence. For example, it has been suggested that abnormal triplex conformation, such as sticky DNA adopted by GAA repeat expansion and/or RNA•DNA hybrids, might impede the transcription of the *FXN* gene^24,25^. In addition, several previous studies of FRDA mouse models, human tissues and cells have highlighted the possibility of epigenetic changes being also either directly or indirectly involved in *FXN* gene silencing^12^. Significant enrichment of the repressive histone modifications at the 5′UTR of the *FXN* gene, together with elevated levels of the non-coding RNA (*FAST-1*) and hypermethylation of CpG regions upstream of the GAA repeat are the epigenetic changes that have been identified in FRDA^10,11,26^. Enrichment of these repressive chromatin marks in the FRDA alleles provides evidence of the heterochromatin formation in the promoter region, involving the critical +1 nucleosome and/or the first intron of the *FXN* gene. Bidichandani and colleagues showed that in FRDA patient-derived fibroblasts, the *FXN* Antisense Transcript (*FAST-1*) was expressed at higher levels. Previous *in vitro* studies in our lab also detected high levels of *FAST-1* in the YG8R and YG22R-derived fibroblast cells^27^. In addition, it has been reported that higher levels of *FAST-1* were associated with the severe CTCF depletion and coincidentally heterochromatin formation in the 5′UTR of the *FXN* gene^14^. It is important to note that FRDA cells do not have a generalized defect in CTCF binding^14^. Our results from overexpressing *FAST-1* in three different non-FRDA cell lines now demonstrate that *FAST-1* can act *in trans* in a similar manner to the *cis-*acting *FAST-1* overexpression that has previously been identified in FRDA fibroblasts. This mechanism of action, where an antisense RNA acts both in *cis* and *trans* and induces epigenetic silencing of its partner sense gene, has been reported for the *p15* and huntingtin (HTT) naturally occurring antisense transcripts^21,28^. We also observed that *FAST-1* overexpressing HeLa cells have a reduced occupancy of CTCF, a decreased level of H3K9ac and increased levels of H3K9me3 at the 5′UTR of the *FXN* gene. This is interesting, because the start of *FAST-1* transcription overlaps with the CTCF binding site in the 5′UTR of the *FXN* gene. The effects of *FAST-1* overexpression on CTCF, H3K9ac and H3K9me3 at the *FXN* 5′UTR locus indicated a potential causative role for *FAST-1* in FRDA.

**Figure 10:**
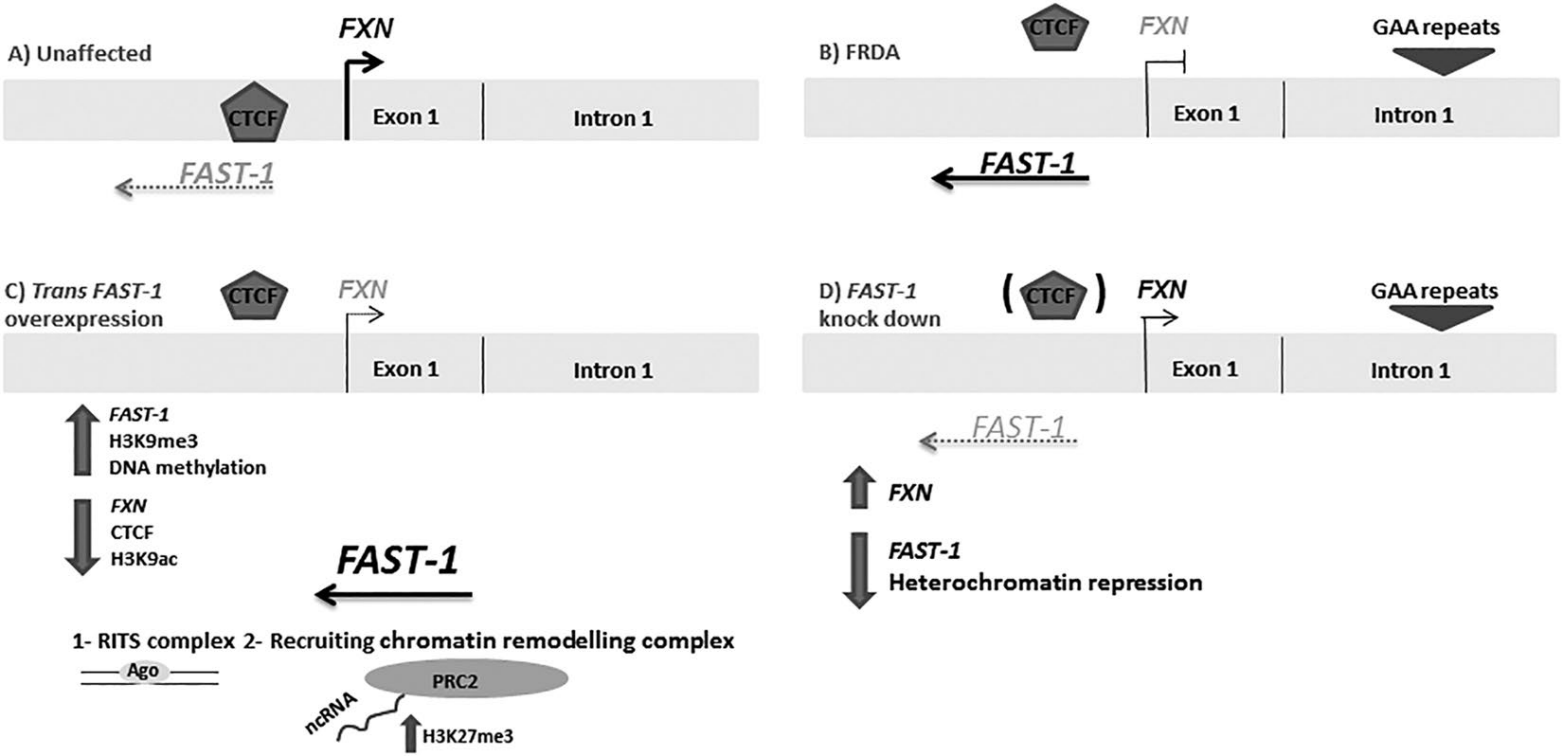
Role of *FAST-1* in FRDA. (**A**) In unaffected individuals, the *FXN* expression is normal, *FAST-1* expression level is low and CTCF is bound to its binding site at 5′UTR of the *FXN* gene. (**B**) In FRDA patients, *FAST-1* expression is elevated and CTCF is depleted from the 5′UTR. (**C**) Following *FAST-1* overexpression, the *FXN* gene and protein expression were decreased. *FAST-1* overexpressing HeLa cells showed reduced occupancy of CTCF, a decreased level of H3K9ac and an increased level of H3K9me3- a hallmark of silenced chromatin- at the 5′UTR of the *FXN* gene. It is plausible to speculate that *FAST-1* overexpression through yet unknown mechanisms (RITS, PRC2) triggers heterochromatin formation and subsequently displaces CTCF from 5′UTR of the *FXN* gene. (**D**) Knocking down *FAST-1* in FRDA fibroblast cells resulted in a 1.5-fold increase in the *FXN* gene expression. Knocking down *FAST-1* may possibly reduce the heterochromatin repression leading to increase in frataxin expression.

Thus, it is plausible that *FAST-1* overexpression may displace CTCF from the 5′UTR of the *FXN* gene, which would result in secondary changes in chromatin structure that are incompatible with CTCF binding to DNA (Fig. 10)^29,30^. It is well established that it is the intrinsic property of the expanded GAA repeat that causes heterochromatin formation and exert its gene silencing effect^9^. It is not clear yet how GAA repeat expansion results in CTCF dislodgement. The expanded GAA repeat drives chromatin changes in intron 1 and this repeat-proximal heterochromatin spreads from the expanded GAA in intron 1 to the upstream regions of the *FXN* gene encompassing the *FXN* promoter and CTCF binding site^6,10^. One possibility is that the heterochromatin emanating from the expanded GAA repeat displaces CTCF from its binding site. Considering the reduced occupancy of CTCF, H3K9me3 enrichment and decreased H3K9ac levels, all hallmarks of heterochromatin formation, at the 5′UTR of the *FXN* gene in *FAST-1* overexpressing HeLa cells, it can be speculated that *FAST-1* RNA, via subsequent heterochromatin, may indirectly displace CTCF. Indeed, several studies have reported that association of H3K9me3 with loss of CTCF binding is a common event in epigenetic silencing of cancer-related genes such as *p16* and *p53*^31,32^. When the chromosomal boundaries are destabilised through dissociation of CTCF and long-range epigenetic organisations are lost, repressive chromatin can spread passively into the promoter region of the *FXN* gene and inhibit its transcription^14,31^. In addition, given the enrichment of CTCF at the border of lamina-associated domains (LADs), loss of CTCF can affect the position of genomic loci relative to the nuclear lamina (NL), a location with a generally repressive environment^33,34^. With this in mind, it has been reported that the majority of expanded *FXN* alleles are positioned at the NL. Thus, CTCF loss and subsequent heterochromatin formation may contribute to *FXN* relocation to the NL and its repression^35^. Recent studies suggest that CTCF has a contradictory function to its classical enhancer-blocker function. It has been proposed that CTCF can tether distant chromatin sites together, create a loop and physically bridge enhancer-promoter interaction^36,37^. In addition, it has been demonstrated that gene looping plays an important role in restricting divergent transcription of non-coding RNA. It can be speculated that CTCF may facilitate enhancer-promoter interactions at the 5′UTR of the *FXN* gene to drive the *FXN* expression. *FAST-1* overexpression and resultant local depletion of CTCF could abolish this CTCF-mediated enhancer-promoter interaction and consequently reduce *FXN* gene expression. Non-coding RNAs have also been found to interact with transcription factors and regulate local gene expression. Two transcription factors, SRF and TFAP2, have been found to regulate expression of the *FXN* gene. They bind directly to a region that is located close to the start of *FAST-1* antisense transcript^38^. Therefore, it is possible that *FAST-1* acts as a decoy, reducing the availability of these transcription factors required for the *FXN* gene transcription.

Antisense transcription is a very common phenomenon in mammalian transcriptomes. Although NATs can show mRNA-like characteristics such as having a poly A tail and a 5′ cap structure, the function of these transcripts is not well understood^39^. Experimental evidence suggests that non-coding RNA can influence gene expression by recruiting chromatin remodelling complexes to specific alleles and mediating heterochromatin formation and gene silencing. It has been shown that the polycomb repressive complex 2 (PRC2) associates with almost 3000 NATs in mouse embryonic stem cells^40,41^. Indeed, antisense transcripts can act as scaffolds or directly recruit PRC2 to chromatin in *cis* and *trans* manners to induce trimethylation of H3K27 and consequently establish a silent chromatin state. According to this mechanism, even a relatively low abundance of antisense transcript can mediate transcriptional repression^42^. PRC2 has also been reported to bind to ncRNA molecules in a size-dependent manner, predominantly around TSSs to maintain the repressed chromatin state^43,44^. In addition, recent studies suggest that there is a cooperative mechanism between H3K27 and H3K9 methylation marks, where H3K9me3 can crosstalk with the Polycomb H3K27me3 modification to cooperate in gene silencing. Indeed, H3K27me3-bound PRC2 stabilizes H3K9me3-anchored HP1 and reinforces heterochromatin formation and ensuing gene repression. Significant enrichment of H3K9 and H3K27 methylation and HP1 has been reported at the *FXN* locus of FRDA-derived fibroblast cells. Here in *FAST-1* overexpressing HeLa cells, we also identified significant enrichment of H3K9me3 at the 5′UTR of the *FXN* gene. Moreover, knowing that genomic repeats are targets of Polycomb complexes and that repeat sequences might provide a binding platform for polycomb group proteins, it is conceivable to speculate that the expanded GAA repeat and high levels of *FAST-1* either directly or indirectly recruit PRC2, and through a cooperative mechanism dictate heterochromatin formation and *FXN* gene silencing in FRDA^19,45^. Alternatively, overexpression of *FAST-1* could be leading to the generation of endogenous short RNAs (endo-siRNAs) thus leading to promotion of the RNA interference pathway^46^.

Our results show that knocking down *FAST-1* in FRDA fibroblast cells increases *FXN* gene expression (Fig. 10). Therefore, it can be concluded that, since *FAST-1* is associated with epigenetic repression of the *FXN* gene, inhibition of *FAST-1* may be an approach to increase the *FXN* transcripts and stimulate subsequent protein expression. Indeed, our results demonstrate that knocking down *FAST-1* in FRDA results in a significant increase in aconitase enzyme activity, a good indicator of frataxin function within cells. Our data suggest that since *FAST-1* is associated with *FXN* gene silencing, inhibition of *FAST-1* may be an approach for FRDA therapy. Considering the nature of NATs and the fact that many currently available drugs would not affect the activity of non-coding RNA molecules, developing new methods to disrupt the function of NATs seems necessary.

## Methods

### Characterization of antisense *FAST-1* transcripts by rapid amplification of cDNA ends (RACE)

RACE experiments were done with DNase I-treated RNA from human FRDA fibroblast cell line (GM04078); 5′- and 3′- RACE were carried out by using the SMARTerTM RACE cDNA amplification kit (Clontech). The gene- specific primers used in each case are:

RACE-FAST-F2 5′-GACCTCCAAGCTTTGCCTCCCTCAAG-3′.

RACE-FAST-R1 5′-GCACCCACTTCCCAGCAAGACAGCAG-3′.

Following the primary *FAST-1* RACE PCR, nested PCRs were performed using the NUP primer (nested universal primer A, supplied with the SMARTer RACE cDNA amplification kit) and a gene specific primer:

RACE-N-FAST-R2 5′-GACAGCAGCTCCCAAGTTCCTCCTG-3′ for the 5′nested PCR.

RACE-N-FAST-F1 5′-GACCCAAGGGAGACTGCAGCCTGGTG-3′ for the 3′nested PCR.

The PCR products were gel purified and cloned for the sequencing analysis.

### *FAST-1* overexpression

Initially, a full-length *FAST-1* sequence (523 bp) was cloned into the expression plasmid pcDNA3. One day before transfection, HeLa, HEK293 and fibroblast cells were seeded in 6 well plates and grown in DMEM containing 10% FBS and antibiotics (penicillin/streptomycin). Two micrograms each of pcDNA3 empty vector and pcDNA3-*FAST-1* (Addgene) were transfected into cells by using Lipofectamine 3000 (Life Technologies) and Neon™ transfection system. After 48 h, 200, 400 and 100 μg/ml of G418 (Gibco) were added to the medium of HeLa, HEK293 and fibroblast cells, respectively, to select for cells carrying the plasmid. After a further 16 days G418-resistant colonies were picked for individual culture, and were maintained in complete medium (DMEM medium containing 10% fetal calf serum) with appropriate G418 concentration.

### Quantitative RT-PCR

RNA was isolated from cells using the Trizol (Invitrogen) method. Following DNase treatment, *FAST-1* cDNA was obtained using the Cloned AMV First-Strand cDNA Synthesis Kit (Invitrogen) with the strand specific FAST RT primer (5′-CCAAGCAGCCTCAATTTGTG-3′). For *FXN* and *HPRT* quantifications, cDNA was synthesised using the oligo (dT)_20_ primer.

Levels of *FAST-1* and *FXN* mRNA expression were assessed by quantitative RT–PCR using a QuantStudio 7 Flex Real-Time PCR instrument and SYBR^®^ Green (Applied Biosystems) with the following primers:

N-FAST F2- forward 5′-GACCCAAGGGAGACTGCAG-3′ and

FAST-R1 -reverse 5′-CACTTCCCAGCAAGACAGC-3′

FXN-h–forward 5′-CAGAGGAAACGCTGGACTCT-3′ and

FXN-h-reverse 5′-AGCCAGATTTGCTTGTTTGGC-3′

HPRT-h-forward 5′-GGTGAAAAGGACCCCACGA-3′and

HPRT-h-reverse 5′-TCAAGGGCATATCCTACAACA-3′

The mRNA expression levels of *HPRT* were also measured in all samples to normalise the gene expression levels to avoid sample-to-sample differences in RNA input, RNA quality and reverse transcription efficiency. Power SYBR® Green Master Mix (Applied Biosystems) was used along with 2 µl of sample cDNA in 10 µl reaction mixture. PCR conditions were set as10 min at 95 °C for enzyme activation followed by 40 two-step cycles (15 sec at 95 °C and 1 min at 60 °C). Reactions were carried out in triplicate for each biological sample and each experiment was repeated at least two times. Values were expressed relative to *HPRT* and expression levels were calculated by 2^−ΔΔCt^ method and RQ manager software (Applied Biosystems).

### Frataxin dipstick assay

Protein concentration was quantified by BCA assay and the levels of frataxin protein were measured by lateral flow immunoassay with the Frataxin Protein Quantity Dipstick Assay Kit (MitoSciences, Eugene, OR, USA) according to the manufacturer’s instructions^47^. Signal intensity was measured with a Hamamatsu ICA-1000 Immunochromatographic Reader (MitoSciences).

### *FAST-1* copy number assay

To detect the number of *FAST-1* copies in *FAST-1* overexpressing cell lines, a custom TaqMan® copy number assay was designed by Life Technologies with a specific TaqMan® FAM™ dye-labeled MGB probe. In brief, 20 ng of genomic DNA was combined with 2 × TaqMan universal master mix, custom designed TaqMan® copy number assay for detecting *FAST-1*, and TaqMan copy number reference assay for RNase P in a 20 μl reaction volume. The assay was performed using the QuantStudio 7 Flex Real-Time PCR instrument (Applied Biosystems) and the following thermal cycling conditions: 50 °C for 2 minutes, 95 °C for 10 minutes, and 40 cycles of 95 °C for 15 seconds and 60 °C for 1 minute. Samples were assayed using triplicate wells for each gene of interest and copy numbers were estimated by relative quantitation (RQ) normalised to the known copy number of the reference sequence using the comparative Ct (ΔΔCt) method. The Ct data were subsequently compared to a calibrator sample of non-transfected control HeLa cells containing two copies of the target sequence, analysed by Applied Biosystems CopyCaller Software (v.2.0; Applied Biosystems) according to the product instruction.

### Chromatin immunoprecipitation-qPCR assay

Histone modifications and CTCF occupancy levels at the 5′UTR of *FXN* gene were detected by ChIP analysis of *FAST-1* overexpressing HeLa cells. This procedure was performed by using ChIP qPCR kit (Chromatrap) with an acetylated H3 (Lys9) (Upstate, 7-352), trimethyl-H3 (Lys9) (Upstate, 07-442) and CTCF (Upstate, 7-729), antibody on formaldehyde cross-linked samples.

DNA was then sheared by sonication, followed by immunoprecipitation. For each experiment, normal rabbit serum (SIGMA) was used as a negative control. After reversal of cross-linking, quantitative RT–PCR amplification of the resultant co-immunoprecipitated DNA was carried out with SYBR® Green in a QuantStudio 7 Flex Real-Time PCR instrument (Applied Biosystems) using the following primers;

FXN-ChIP-forward 5′-TCCTGAGGTCTAACCTCTAGCTGC-3′ and

FXN-ChIP-reverse 5′-CGAGAGTCCACATGCTGCTCC-3′

GAPDH-ChIP-forward 5′-TCGACAGTCAGCCGCATCT-3′ and

GAPDH-ChIP-reverse 5′-CTAGCCTCCCGGGTTTCTCT-3′

FXN-pro-forward 5′- CCCCACATACCCAACTGCTG-3′and

FXN-pro-reverse 5′-GCCCGCCGCTTCTAAAATTC-3′

The data were from three independent chromatin preparations, with each experiment done in triplicate.

### MethylScreen assay

DNA methylation analysis was performed using the ‘MethylScreen’ method^19^, which uses combined restriction digestion of DNA with methylation sensitive and methylation dependent restriction enzymes, MSRE and MDRE respectively. MethylScreen was used to analyse the two CpG sites, CpG6 and CpG11^11^, at *FXN* locus upstream of the GAA repeat. 1 µg of genomic DNA was digested with: (1) a MSRE, (2) MDRE, (3) both MSRE and MDRE (double digest, DD), and (4) neither MSRE or MDRE (mock control). The MSREs used for CpGs 6 and 11 were *Aji*I (Fermentas) and Hpy188III (New England Biolabs), respectively^38^. The MDRE used for all two CpGs was McrBc (Fermentas). A 50 ng aliquot of digested DNA was then amplified by quantitative PCR using SYBR® Green (Applied Biosystems) and an ABI 7900 Fast Real-Time PCR System (Applied Biosystems) with the following primers: CpG6 F 5′-GAAGATGCCAAGGAAGTGGTAG-3′ and R 5′-GAGCAACACAAATATGGCTTGG-3′; CpG11 F 5′-GATCCGTCTGGGCAAAGGCCAG-3′ and R 5′-ATCCCAAAGTTTCTTCAAACACAATG-3′. PCR quantification was carried out using the ΔCt method (values were calculated as 2^ΔCt^ (mock – digest) with the mock value set at 100%) and RQ Manager Software (Applied Biosystems). Each qRT-PCR reaction was performed in triplicate. MethylScreen DNA methylation values were then calculated as follows: Densely methylated (DM) = (MSRE − DD)/(100 − DD) × 100; unmethylated (UM) = (MDRE − DD)/(100 − DD) × 100; intermediately methylated (IM) = 100–(DM + UM).

### Knocking down *FAST-1* expression

To knock down *FAST-1* expression, Sigma custom cloning MISSION shRNA team designed a short hairpin RNA, cloned it into a pLKO.1-puro-CMV-tGFP vector and provided us with ready to use lentiviral particles for transduction. To achieve a stable *FAST-1* knockdown, FRDA and normal fibroblast cell lines were transduced with lentiviral (LV) vectors expressing shRNAs targeting *FAST-1* (pLKO.1-puro-CMV-tGFP). Several precautions were taken in order to control off-target effects and specificity, as follows: (i) pLKO.1-puro Non-Target shRNA was used as the negative control; (ii) The normal fibroblast cell line was also transduced with LV *FAST-1* to assess the specificity of any *FAST-1* knockdown effect on FRDA cells. Briefly, cells were seeded into 6-well plates with culture medium containing 10% FBS until 80% confluence, and the cells were then incubated with *FAST-1* and scrambled LV particles (25ul–100ul of 5.7 × 10^7^ to achieve MOI values of 5–20) in serum-free culture medium supplemented with polybrene (8 μg/ml) for 6 h. Then the supernatant was replaced with 1 ml fresh medium and incubated at 37 °C and 5% CO_2_ for 72 hours to allow time for the expression of the reporter and puromycin resistance genes. After 72 hours, the selective antibiotic, puromycin, was added to the media at a final concentration of 0.2 μg/μl. The selection media was replaced every 3 days and the final cell harvest for RNA analysis was performed at 2 weeks post-transduction.

### Aconitase assay

Aconitase activities were determined using the Aconitase Assay Kit (Cayman Chemical Company, 705502). To perform the assays, cultured cells were washed with cold PBS, scraped off the flask and pelleted by centrifugation at 800 g for 10 min. Cell pellets were suspended in cell lytic buffer (Cayman) for 15 min at 4 °C, then centrifuged at 10,000 g for 10 min, followed by BCA protein concentration determination and dilution of cell protein lysates to 0.5ug/µl in 1x assay buffer (Cayman). 50 µl of 0.5 ug/µl cell protein lysates were added to 200 µl of substrate mix (50 mM Tris/HCl pH 7.4, 0.4 mM NADP, 5 mM Na citrate, 0.6 mM MgCl_2_, 0.1% (v/v) Triton X-100 and 1 U isocitrate dehydrogenase) and the reactions were incubated at 37 °C for 15 min, followed by spectrophotometric 340 nm absorbance measurements every minute for 15 min at 37 °C to determine the reaction slope. Aconitase activities of samples were then normalized to citrate synthase activities, which were determined using a citrate synthase assay kit (Sigma, CS0720).

### Statistical analysis

Statistical analyses that compared two groups of data were performed using the Student’s t test, with a P value of <0.05 taken to indicate a statistically significant difference. Correlation and regression analyses of *FAST-1* copy number, *FAST-1* expression and *FXN* expression were performed using Microsoft Excel data analysis tools.

## Data Availability

All data generated or analysed during this study are included in this published article.
